# Methano­ldioxido{1-[(2*RS*)-(2-oxidoprop­yl)iminometh­yl]-2-naphtholato}molybdenium(VI)

**DOI:** 10.1107/S160053681000262X

**Published:** 2010-01-27

**Authors:** Monadi Niaz, Sheikhshoaie Iran, Rezaeifard Abdolreza

**Affiliations:** a Chemistry Department, Shahid Bahonar University of Kerman, Kerman, Iran; b Shahid Bahonar University of Kerman, Kerman, Iran; c Birjand University, Birjand, Iran

## Abstract

Crystals of the title compound, [Mo(C_14_H_13_NO_2_)O_2_(CH_4_O)], were obtained by recrystallization from methanol. The Mo^VI^ atom is coordinated by two oxide O atoms and by two O atoms and one N atom of the tridentate 1-[(2-oxidoprop­yl)iminometh­yl]-2-naphtholate Schiff base ligand. The coordination sphere is completed by the O atom of a methanol mol­ecule, yielding a distorted octa­hedron. O—H⋯O hydrogen bonding yields centrosymmetric dimers.

## Related literature

For related structures with O= Mo^VI^=O units and for the synthesis, see: Arnaiz *et al.* (2000 ▶); Holm *et al.* (1996 ▶); Syamal & Maurya (1989 ▶). For the prperties of related compounds, see: Arnold *et al.* (2001 ▶); Bagherzadeh *et al.* (2009 ▶); Bruno *et al.* (2006 ▶); Holm (1987 ▶); Maurya *et al.* (1997 ▶); Schurig & Betschinger (1992 ▶); Sheikhshoaie *et al.* (2009 ▶).
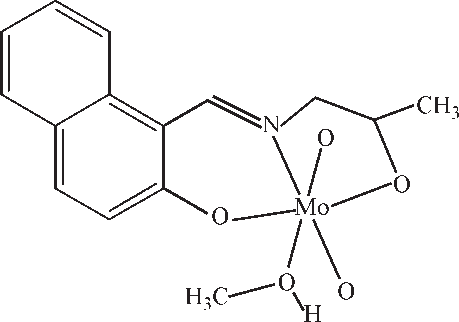


## Experimental

### 

#### Crystal data

[Mo(C_14_H_13_NO_2_)O_2_(CH_4_O)]
*M* *_r_* = 387.24
Monoclinic, 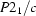

*a* = 7.9064 (5) Å
*b* = 15.078 (1) Å
*c* = 12.6796 (8) Å
β = 93.959 (1)°
*V* = 1507.96 (17) Å^3^
*Z* = 4
Mo *K*α radiation
μ = 0.89 mm^−1^
*T* = 100 K
0.19 × 0.16 × 0.16 mm

#### Data collection

Bruker APEXII CCD area-detector diffractometer
Absorption correction: multi-scan (*SADABS*; Sheldrick, 1996 ▶) *T*_min_ = 0.849, *T*_max_ = 0.870
18948 measured reflections
4393 independent reflections
3951 reflections with *I* > 2σ(*I*)
*R*_int_ = 0.026

#### Refinement

*R*[*F* ^2^ > 2σ(*F* ^2^)] = 0.023
*wR*(*F* ^2^) = 0.056
*S* = 1.01
4393 reflections
202 parameters
H-atom parameters constrained
Δρ_max_ = 0.48 e Å^−3^
Δρ_min_ = −0.65 e Å^−3^

### 

Data collection: *APEX2* (Bruker, 2007 ▶); cell refinement: *SAINT* (Bruker, 2007 ▶); data reduction: *SAINT*; program(s) used to solve structure: *SHELXS97* (Sheldrick, 2008 ▶); program(s) used to refine structure: *SHELXL97* (Sheldrick, 2008 ▶); molecular graphics: *SHELXTL* (Sheldrick, 2008 ▶); software used to prepare material for publication: *SHELXTL*.

## Supplementary Material

Crystal structure: contains datablocks I, global. DOI: 10.1107/S160053681000262X/fi2095sup1.cif

Structure factors: contains datablocks I. DOI: 10.1107/S160053681000262X/fi2095Isup2.hkl

Additional supplementary materials:  crystallographic information; 3D view; checkCIF report

## Figures and Tables

**Table 1:** Hydrogen-bond geometry (Å, °)

*D*—H⋯*A*	*D*—H	H⋯*A*	*D*⋯*A*	*D*—H⋯*A*
O5—H5*O*⋯O2^i^	0.85	1.82	2.6667 (16)	179

Symmetry code: (i) 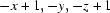.

**Fig. 1.**
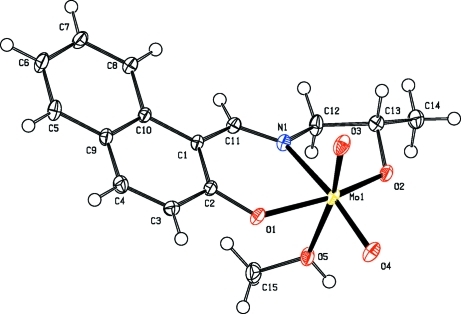
The molecular structure of the title compound. Thermal ellispoids at the 50% probability level.

**Fig. 2.**
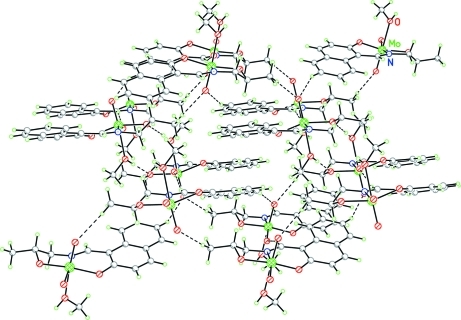
The hydrogen bonding pattern in the title compound yielding centrosymmetric dimers. H bonds indicated by dashed lines. Moiety to the left generated by (i) 1–x, –y, 1–z.

## supplementary crystallographic information

### Comment

Transition metal oxo compounds containing Schiff base ligands have been in the focus of scientific interest for many years. These compounds are involved in oxygen transfer chemistry in both biological and industrial processes (Maurya *et al.*, 1997), effective catalysts for epoxidation (Bagherzadeh *et al.*,  2009; Holm, 1987; Schurig & Betschinger, 1992; Arnold *et al.*, 2001). The success of molybdenum(VI) complexes in reactions to produce racemic epoxides led to the belief that some derivatives of these complexes could be applied as chiral catalysts (Bruno *et al.*, 2006), and oxidation catalysis (Sheikhshoaie *et al.*, 2009). Continuing our interest in the structural chemistry of dioxomolybdenum(VI) Schiff base complexes, we have synthesized and structurally characterized the title complex.

The molecular structure of the title complex is illustrated in Figure 1. The Mo^VI^ ion is in a distored octahedral environment being coordinated by two oxido O atoms (O4 and O3), three atoms (two oxygen and one nitrogen atoms) of the tridentate Schiff base ligand and one oxygen atom from methanol. The oxido-O atoms are in cis position with short Mo=O bonds (1.7001 (12) and 1.7140 (12)Å, respectively). The OH group of the methanol molecule acts as H bond donor, yielding centrosymmetric dimers (Fig. 2).

### Experimental

To a solution of 0.229 mg (1 mmol) of tridentate Schiff base ligand 1-((*E*)-(2-hydroxypropylimino)methyl)naphthalen-2-ol) in 15 ml dry methanol was added a solution of 0.327 mg (1 mmol) of MoO2(acac)2 in 10 ml dry methanol, and refluxed for an additional 2 h. {[(1-amino-2-hydroxypropane)nitilomethylidyne-(2-naphthalato)]-dioxidomolybdenum(VI)(Methanol)} was obtained as a yellow microcrystalline precipitate. The precipitate was filtered off, washed with 5 ml absolute ethanol. Small yellow crystals formed upon recrystallisation from methanol.

### Refinement

The hydrogen atoms of OH group was found in difference Fourier synthesis. The H(C) atom positions were calculated. All hydrogen atoms were refined in isotropic approximation in riding model, the Uiso(H) parameters were fixed to 1.2 Ueq(Ci), for methyl groups to 1.5 Ueq(Cii), where U(Ci) and U(Cii) are respectively the equivalent thermal parameters of the carbon atoms to which corresponding H atoms are bonded

### Crystal data

**Table:**

[Mo(C_14_H_13_NO_2_)O_2_(CH_4_O)]	*F*(000) = 784
*M**_r_* = 387.24	*D*_x_ = 1.706 Mg m^−^^3^
Monoclinic, *P*2_1_/*c*	Mo *K*α radiation, λ = 0.71073 Å
Hall symbol: -P 2ybc	Cell parameters from 211 reflections
*a* = 7.9064 (5) Å	θ = 3–30°
*b* = 15.078 (1) Å	µ = 0.89 mm^−^^1^
*c* = 12.6796 (8) Å	*T* = 100 K
β = 93.959 (1)°	Prism, pale yellow
*V* = 1507.96 (17) Å^3^	0.19 × 0.16 × 0.16 mm
*Z* = 4	

### Data collection

**Table:**

Bruker APEXII CCD area-detector diffractometer	4393 independent reflections
Radiation source: fine-focus sealed tube	3951 reflections with *I* > 2σ(*I*)
graphite	*R*_int_ = 0.026
ω scans	θ_max_ = 30.0°, θ_min_ = 2.1°
Absorption correction: multi-scan (*SADABS*; Sheldrick, 1996)	*h* = −11→11
*T*_min_ = 0.849, *T*_max_ = 0.870	*k* = −21→21
18948 measured reflections	*l* = −17→17

### Refinement

**Table:**

Refinement on *F*^2^	Primary atom site location: structure-invariant direct methods
Least-squares matrix: full	Secondary atom site location: structure-invariant direct methods
*R*[*F*^2^ > 2σ(*F*^2^)] = 0.023	Hydrogen site location: inferred from neighbouring sites
*wR*(*F*^2^) = 0.056	H-atom parameters constrained
*S* = 1.01	*w* = 1/[σ^2^(*F*_o_^2^) + (0.0248*P*)^2^ + 1.35*P*] where *P* = (*F*_o_^2^ + 2*F*_c_^2^)/3
4393 reflections	(Δ/σ)_max_ = 0.008
202 parameters	Δρ_max_ = 0.48 e Å^−^^3^
0 restraints	Δρ_min_ = −0.65 e Å^−^^3^

### Special details

**Table:**

Geometry. All e.s.d.'s (except the e.s.d. in the dihedral angle between two l.s. planes) are estimated using the full covariance matrix. The cell e.s.d.'s are taken into account individually in the estimation of e.s.d.'s in distances, angles and torsion angles; correlations between e.s.d.'s in cell parameters are only used when they are defined by crystal symmetry. An approximate (isotropic) treatment of cell e.s.d.'s is used for estimating e.s.d.'s involving l.s. planes.
Refinement. Refinement of *F*^2^ against ALL reflections. The weighted *R*-factor *wR* and goodness of fit *S* are based on *F*^2^, conventional *R*-factors *R* are based on *F*, with *F* set to zero for negative *F*^2^. The threshold expression of *F*^2^ > σ(*F*^2^) is used only for calculating *R*-factors(gt) *etc*. and is not relevant to the choice of reflections for refinement. *R*-factors based on *F*^2^ are statistically about twice as large as those based on *F*, and *R*- factors based on ALL data will be even larger.

### Fractional atomic coordinates and isotropic or equivalent isotropic displacement parameters (Å^2^)

**Table:**

	*x*	*y*	*z*	*U*_iso_*/*U*_eq_	
Mo1	0.530764 (16)	0.118564 (9)	0.344992 (10)	0.01228 (4)	
O1	0.58279 (14)	0.07661 (8)	0.20452 (9)	0.0154 (2)	
O2	0.58637 (14)	0.12750 (7)	0.49712 (9)	0.0149 (2)	
O3	0.51942 (15)	0.22791 (8)	0.31257 (10)	0.0203 (2)	
O4	0.32656 (14)	0.08304 (9)	0.35495 (10)	0.0197 (2)	
O5	0.60141 (16)	−0.02912 (8)	0.38095 (10)	0.0191 (2)	
H5O	0.5424	−0.0612	0.4195	0.029 (6)*	
N1	0.81591 (16)	0.11919 (9)	0.36213 (10)	0.0132 (2)	
C1	0.86943 (19)	0.12061 (10)	0.17666 (12)	0.0122 (3)	
C2	0.70542 (19)	0.09463 (10)	0.14066 (12)	0.0129 (3)	
C3	0.6632 (2)	0.08250 (11)	0.03095 (13)	0.0157 (3)	
H3A	0.5510	0.0660	0.0072	0.019*	
C4	0.7821 (2)	0.09422 (11)	−0.04064 (13)	0.0178 (3)	
H4A	0.7531	0.0827	−0.1133	0.021*	
C5	1.0683 (2)	0.14082 (12)	−0.08389 (13)	0.0197 (3)	
H5A	1.0391	0.1293	−0.1565	0.024*	
C6	1.2258 (2)	0.17414 (12)	−0.05370 (14)	0.0219 (3)	
H6A	1.3049	0.1859	−0.1051	0.026*	
C7	1.2691 (2)	0.19074 (11)	0.05381 (14)	0.0191 (3)	
H7A	1.3777	0.2143	0.0748	0.023*	
C8	1.15642 (19)	0.17334 (11)	0.12916 (13)	0.0156 (3)	
H8A	1.1883	0.1850	0.2014	0.019*	
C9	0.9488 (2)	0.12333 (11)	−0.00845 (12)	0.0152 (3)	
C10	0.99294 (19)	0.13813 (10)	0.10045 (12)	0.0126 (3)	
C11	0.91962 (19)	0.12357 (10)	0.28859 (12)	0.0138 (3)	
H11A	1.0370	0.1291	0.3090	0.017*	
C12	0.8805 (2)	0.11752 (12)	0.47316 (12)	0.0180 (3)	
H12A	0.9898	0.1496	0.4824	0.022*	
H12B	0.8983	0.0556	0.4975	0.022*	
C13	0.7473 (2)	0.16280 (11)	0.53555 (13)	0.0171 (3)	
H13A	0.7493	0.2280	0.5215	0.021*	
C14	0.7770 (2)	0.14687 (12)	0.65327 (13)	0.0207 (3)	
H14A	0.6881	0.1765	0.6904	0.031*	
H14B	0.8879	0.1707	0.6782	0.031*	
H14C	0.7741	0.0830	0.6675	0.031*	
C15	0.6958 (2)	−0.08893 (12)	0.32155 (16)	0.0254 (4)	
H15A	0.7596	−0.1296	0.3697	0.038*	
H15B	0.7749	−0.0555	0.2805	0.038*	
H15C	0.6182	−0.1230	0.2735	0.038*	

### Atomic displacement parameters (Å^2^)

**Table:**

	*U*^11^	*U*^22^	*U*^33^	*U*^12^	*U*^13^	*U*^23^
Mo1	0.00938 (6)	0.01464 (7)	0.01329 (7)	0.00158 (5)	0.00413 (4)	0.00367 (5)
O1	0.0115 (5)	0.0216 (6)	0.0134 (5)	−0.0023 (4)	0.0032 (4)	0.0024 (4)
O2	0.0145 (5)	0.0171 (5)	0.0139 (5)	−0.0004 (4)	0.0057 (4)	0.0014 (4)
O3	0.0199 (6)	0.0187 (6)	0.0232 (6)	0.0048 (5)	0.0084 (5)	0.0080 (5)
O4	0.0122 (5)	0.0270 (6)	0.0203 (6)	−0.0003 (5)	0.0038 (4)	0.0053 (5)
O5	0.0249 (6)	0.0135 (5)	0.0205 (6)	0.0016 (5)	0.0130 (5)	0.0023 (4)
N1	0.0117 (6)	0.0159 (6)	0.0121 (6)	0.0016 (5)	0.0018 (4)	0.0015 (5)
C1	0.0120 (6)	0.0139 (7)	0.0110 (6)	0.0004 (5)	0.0033 (5)	0.0007 (5)
C2	0.0122 (6)	0.0129 (7)	0.0138 (7)	0.0002 (5)	0.0040 (5)	0.0010 (5)
C3	0.0150 (7)	0.0166 (7)	0.0155 (7)	−0.0029 (6)	0.0014 (5)	−0.0012 (6)
C4	0.0195 (8)	0.0217 (8)	0.0125 (7)	−0.0011 (6)	0.0024 (6)	−0.0024 (6)
C5	0.0208 (8)	0.0255 (8)	0.0137 (7)	0.0009 (6)	0.0073 (6)	0.0007 (6)
C6	0.0201 (8)	0.0260 (9)	0.0212 (8)	−0.0014 (7)	0.0118 (6)	0.0013 (7)
C7	0.0136 (7)	0.0208 (8)	0.0238 (8)	−0.0018 (6)	0.0074 (6)	0.0007 (6)
C8	0.0127 (7)	0.0180 (7)	0.0164 (7)	−0.0005 (5)	0.0032 (6)	0.0007 (6)
C9	0.0159 (7)	0.0169 (7)	0.0133 (7)	0.0008 (6)	0.0048 (5)	0.0001 (5)
C10	0.0129 (6)	0.0120 (7)	0.0132 (7)	0.0006 (5)	0.0043 (5)	0.0015 (5)
C11	0.0117 (6)	0.0159 (7)	0.0139 (7)	0.0003 (5)	0.0016 (5)	0.0010 (5)
C12	0.0143 (7)	0.0290 (8)	0.0109 (7)	0.0033 (6)	0.0019 (5)	−0.0001 (6)
C13	0.0181 (7)	0.0171 (8)	0.0165 (7)	−0.0018 (6)	0.0044 (6)	−0.0002 (6)
C14	0.0238 (8)	0.0252 (8)	0.0135 (7)	−0.0032 (7)	0.0038 (6)	−0.0015 (6)
C15	0.0293 (9)	0.0179 (8)	0.0310 (10)	0.0028 (7)	0.0161 (8)	−0.0015 (7)

### Geometric parameters (Å, °)

**Table:**

Mo1—O3	1.7001 (12)	C5—C9	1.415 (2)
Mo1—O4	1.7140 (12)	C5—H5A	0.9500
Mo1—O2	1.9533 (11)	C6—C7	1.405 (3)
Mo1—O1	1.9604 (11)	C6—H6A	0.9500
Mo1—N1	2.2500 (13)	C7—C8	1.376 (2)
Mo1—O5	2.3331 (12)	C7—H7A	0.9500
O1—C2	1.3334 (18)	C8—C10	1.421 (2)
O2—C13	1.433 (2)	C8—H8A	0.9500
O5—C15	1.420 (2)	C9—C10	1.418 (2)
O5—H5O	0.8499	C11—H11A	0.9500
N1—C11	1.2852 (19)	C12—C13	1.522 (2)
N1—C12	1.464 (2)	C12—H12A	0.9900
C1—C2	1.401 (2)	C12—H12B	0.9900
C1—C10	1.445 (2)	C13—C14	1.514 (2)
C1—C11	1.448 (2)	C13—H13A	1.0000
C2—C3	1.420 (2)	C14—H14A	0.9800
C3—C4	1.362 (2)	C14—H14B	0.9800
C3—H3A	0.9500	C14—H14C	0.9800
C4—C9	1.422 (2)	C15—H15A	0.9800
C4—H4A	0.9500	C15—H15B	0.9800
C5—C6	1.373 (2)	C15—H15C	0.9800
			
O3—Mo1—O4	106.65 (6)	C7—C6—H6A	120.3
O3—Mo1—O2	100.16 (6)	C8—C7—C6	120.91 (16)
O4—Mo1—O2	95.61 (5)	C8—C7—H7A	119.5
O3—Mo1—O1	95.98 (5)	C6—C7—H7A	119.5
O4—Mo1—O1	102.94 (5)	C7—C8—C10	120.96 (15)
O2—Mo1—O1	150.75 (5)	C7—C8—H8A	119.5
O3—Mo1—N1	93.16 (5)	C10—C8—H8A	119.5
O4—Mo1—N1	159.54 (5)	C5—C9—C10	119.88 (15)
O2—Mo1—N1	75.44 (5)	C5—C9—C4	120.82 (15)
O1—Mo1—N1	79.48 (5)	C10—C9—C4	119.25 (14)
O3—Mo1—O5	168.75 (5)	C9—C10—C8	117.79 (13)
O4—Mo1—O5	84.34 (5)	C9—C10—C1	119.36 (14)
O2—Mo1—O5	80.69 (4)	C8—C10—C1	122.80 (14)
O1—Mo1—O5	78.88 (4)	N1—C11—C1	124.35 (14)
N1—Mo1—O5	76.13 (5)	N1—C11—H11A	117.8
C2—O1—Mo1	133.87 (10)	C1—C11—H11A	117.8
C13—O2—Mo1	119.69 (9)	N1—C12—C13	106.52 (13)
C15—O5—Mo1	129.06 (10)	N1—C12—H12A	110.4
C15—O5—H5O	105.9	C13—C12—H12A	110.4
Mo1—O5—H5O	121.4	N1—C12—H12B	110.4
C11—N1—C12	120.05 (13)	C13—C12—H12B	110.4
C11—N1—Mo1	127.98 (11)	H12A—C12—H12B	108.6
C12—N1—Mo1	111.93 (9)	O2—C13—C14	110.47 (13)
C2—C1—C10	119.11 (14)	O2—C13—C12	106.65 (13)
C2—C1—C11	120.87 (13)	C14—C13—C12	112.07 (14)
C10—C1—C11	119.86 (13)	O2—C13—H13A	109.2
O1—C2—C1	123.69 (14)	C14—C13—H13A	109.2
O1—C2—C3	115.93 (14)	C12—C13—H13A	109.2
C1—C2—C3	120.34 (14)	C13—C14—H14A	109.5
C4—C3—C2	120.66 (15)	C13—C14—H14B	109.5
C4—C3—H3A	119.7	H14A—C14—H14B	109.5
C2—C3—H3A	119.7	C13—C14—H14C	109.5
C3—C4—C9	121.14 (15)	H14A—C14—H14C	109.5
C3—C4—H4A	119.4	H14B—C14—H14C	109.5
C9—C4—H4A	119.4	O5—C15—H15A	109.5
C6—C5—C9	121.00 (16)	O5—C15—H15B	109.5
C6—C5—H5A	119.5	H15A—C15—H15B	109.5
C9—C5—H5A	119.5	O5—C15—H15C	109.5
C5—C6—C7	119.43 (15)	H15A—C15—H15C	109.5
C5—C6—H6A	120.3	H15B—C15—H15C	109.5
			
O3—Mo1—O1—C2	57.22 (14)	O1—C2—C3—C4	−176.28 (15)
O4—Mo1—O1—C2	165.86 (14)	C1—C2—C3—C4	1.3 (2)
O2—Mo1—O1—C2	−66.15 (18)	C2—C3—C4—C9	−3.3 (3)
N1—Mo1—O1—C2	−34.90 (14)	C9—C5—C6—C7	0.3 (3)
O5—Mo1—O1—C2	−112.66 (14)	C5—C6—C7—C8	0.6 (3)
O3—Mo1—O2—C13	−66.97 (11)	C6—C7—C8—C10	0.0 (3)
O4—Mo1—O2—C13	−175.04 (11)	C6—C5—C9—C10	−1.6 (3)
O1—Mo1—O2—C13	55.48 (15)	C6—C5—C9—C4	175.93 (17)
N1—Mo1—O2—C13	23.68 (11)	C3—C4—C9—C5	−175.91 (16)
O5—Mo1—O2—C13	101.65 (11)	C3—C4—C9—C10	1.6 (2)
O3—Mo1—O5—C15	−43.8 (3)	C5—C9—C10—C8	2.1 (2)
O4—Mo1—O5—C15	124.28 (15)	C4—C9—C10—C8	−175.50 (15)
O2—Mo1—O5—C15	−139.07 (15)	C5—C9—C10—C1	179.57 (15)
O1—Mo1—O5—C15	19.88 (15)	C4—C9—C10—C1	2.0 (2)
N1—Mo1—O5—C15	−61.89 (15)	C7—C8—C10—C9	−1.3 (2)
O3—Mo1—N1—C11	−72.74 (14)	C7—C8—C10—C1	−178.68 (15)
O4—Mo1—N1—C11	121.60 (18)	C2—C1—C10—C9	−3.9 (2)
O2—Mo1—N1—C11	−172.43 (14)	C11—C1—C10—C9	171.46 (14)
O1—Mo1—N1—C11	22.76 (13)	C2—C1—C10—C8	173.43 (15)
O5—Mo1—N1—C11	103.77 (14)	C11—C1—C10—C8	−11.2 (2)
O3—Mo1—N1—C12	104.86 (11)	C12—N1—C11—C1	176.40 (15)
O4—Mo1—N1—C12	−60.8 (2)	Mo1—N1—C11—C1	−6.2 (2)
O2—Mo1—N1—C12	5.18 (10)	C2—C1—C11—N1	−13.1 (2)
O1—Mo1—N1—C12	−159.63 (11)	C10—C1—C11—N1	171.56 (15)
O5—Mo1—N1—C12	−78.62 (11)	C11—N1—C12—C13	148.93 (15)
Mo1—O1—C2—C1	29.3 (2)	Mo1—N1—C12—C13	−28.89 (15)
Mo1—O1—C2—C3	−153.24 (12)	Mo1—O2—C13—C14	−168.83 (10)
C10—C1—C2—O1	179.71 (14)	Mo1—O2—C13—C12	−46.80 (15)
C11—C1—C2—O1	4.4 (2)	N1—C12—C13—O2	45.45 (16)
C10—C1—C2—C3	2.3 (2)	N1—C12—C13—C14	166.45 (13)
C11—C1—C2—C3	−173.00 (14)		

### Hydrogen-bond geometry (Å, °)

**Table:**

*D*—H···*A*	*D*—H	H···*A*	*D*···*A*	*D*—H···*A*
O5—H5O···O2^i^	0.85	1.82	2.6667 (16)	179

Symmetry codes: (i) −*x*+1, −*y*, −*z*+1.
